# Correction: A CO_2_-responsive smart fluid based on supramolecular assembly structures varying reversibly from vesicles to wormlike micelles

**DOI:** 10.1039/d0ra90076a

**Published:** 2020-07-21

**Authors:** Chunming Xiong, Falin Wei, Qiang Zhou, Kang Peng, Zhengrong Ye, Haiyang Yang

**Affiliations:** Research Institute of Science and Technology, China National Petroleum Corporation Beijing 100083 P. R. China; CAS Key Laboratory of Soft Matter Chemistry, School of Chemistry and Materials Science, University of Science and Technology of China Hefei 230026 P. R. China yhy@ustc.edu.cn pengkang@mail.ustc.edu.cn

## Abstract

Correction for ‘A CO_2_-responsive smart fluid based on supramolecular assembly structures varying reversibly from vesicles to wormlike micelles’ by Chunming Xiong *et al.*, *RSC Adv.*, 2020, **10**, 25311–25318, DOI: 10.1039/D0RA03854G.

The authors regret that the affiliations were incorrectly shown in the original manuscript. The corrected list of affiliations is as shown above.

The Royal Society of Chemistry apologises for these errors and any consequent inconvenience to authors and readers.

## Supplementary Material

